# Increased cardiac Pi/PCr in the diabetic heart observed using phosphorus magnetic resonance spectroscopy at 7T

**DOI:** 10.1371/journal.pone.0269957

**Published:** 2022-06-16

**Authors:** Ladislav Valkovič, Andrew Apps, Jane Ellis, Stefan Neubauer, Damian J. Tyler, Albrecht Ingo Schmid, Oliver J. Rider, Christopher T. Rodgers

**Affiliations:** 1 Oxford Centre for Clinical MR Research (OCMR), RDM Cardiovascular Medicine, University of Oxford, Oxford, United Kingdom; 2 Institute of Measurement Science, Slovak Academy of Sciences, Bratislava, SVK; 3 Department of Physiology, Anatomy and Genetics, University of Oxford, Oxford, United Kingdom; 4 High Field MR Center, Center for Medical Physics and Biomedical Engineering, Medical University of Vienna, Vienna, AT; 5 Department of Clinical Neurosciences, Wolfson Brain Imaging Centre, University of Cambridge, Cambridge, United Kingdom; Scuola Superiore Sant’Anna, ITALY

## Abstract

Phosphorus magnetic resonance spectroscopy (^31^P-MRS) has previously demonstrated decreased energy reserves in the form of phosphocreatine to adenosine-tri-phosphate ratio (PCr/ATP) in the hearts of patients with type 2 diabetes (T2DM). Recent ^31^P-MRS techniques using 7T systems, e.g. long mixing time stimulated echo acquisition mode (STEAM), allow deeper insight into cardiac metabolism through assessment of inorganic phosphate (Pi) content and myocardial pH, which play pivotal roles in energy production in the heart. Therefore, we aimed to further explore the cardiac metabolic phenotype in T2DM using STEAM at 7T. Seventeen patients with T2DM and twenty-three healthy controls were recruited and their cardiac PCr/ATP, Pi/PCr and pH were assessed at 7T. Diastolic function of all patients with T2DM was assessed using echocardiography to investigate the relationship between diastolic dysfunction and cardiac metabolism. Mirroring the decreased PCr/ATP (1.70±0.31 vs. 2.07±0.39; p<0.01), the cardiac Pi/PCr was increased (0.13±0.07 vs. 0.10±0.03; p = 0.02) in T2DM patients in comparison to healthy controls. Myocardial pH was not significantly different between the groups (7.14±0.12 vs. 7.10±0.12; p = 0.31). There was a negative correlation between PCr/ATP and diastolic function (R^2^ = 0.33; p = 0.02) in T2DM. No correlation was observed between diastolic function and Pi/PCr and (R^2^ = 0.16; p = 0.21). In addition, we did not observe any correlation between cardiac PCr/ATP and Pi/PCr (p = 0.19). Using STEAM ^31^P-MRS at 7T we have for the first time explored Pi/PCr in the diabetic human heart and found it increased when compared to healthy controls. The lack of correlation between measured PCr/ATP and Pi/PCr suggests that independent mechanisms might contribute to these perturbations.

## Introduction

Type 2 diabetes mellitus (T2DM) is a global health concern and the cardiac consequences of T2DM are numerous. Their development is typically through an asymptomatic phase of diastolic dysfunction [1], which culminates in diabetic cardiomyopathy, often defined as the onset of heart failure in the absence of significant coronary artery disease, hypertension, or valvular heart disease [2,3]. The presence of diastolic dysfunction doubles the five year risk of heart failure in T2DM [3]. Hence, there is a growing need to better understand the molecular mechanisms contributing to these processes [4] and to develop non-invasive techniques to assess them.

In T2DM there is an increasingly recognised pathological metabolic phenotype [5]. As the heart requires a vast amount of adenosine-triphosphate (ATP) to maintain both contraction and relaxation, and ATP metabolism is directly linked to changes in function, this is not surprising. Of particular importance is the pathological switch in cardiac substrate preference [6], i.e., upregulated β-oxidation of fatty acids and reduced myocardial uptake and oxidation of glucose [7], which inhibits pyruvate dehydrogenase flux [8,9] and results in reduced efficiency of ATP production and impaired creatine kinase (CK) flux.

Phosphorus magnetic resonance spectroscopy (^31^P-MRS) allows non-invasive assessment of high-energy phosphate metabolites, e.g., ATP and phosphocreatine (PCr), and hence allows tissue metabolism assessment in vivo [10–12]. A reduced cardiac PCr/ATP has been shown in several metabolic diseases including T2DM [4] and obesity [13]. However, further insight into the metabolic state of the heart could be gained by assessing the cytosolic content of inorganic phosphate (Pi), which has a strong impact on the Gibbs energy of ATP hydrolysis. Quantifying myocardial Pi in the diabetic heart may, therefore, help explain latent diastolic dysfunction.

Until recently, cardiac Pi levels have been difficult to quantify due to strong overlapping signals originating in the circulating blood. Myocardial Pi can, however, be robustly quantified using ultra-high fields, i.e. 7T. Unlike the energy absorption-heavy decoupling applied at 1.5T [14], long TR acquisition can be used, exploiting the relaxation properties of Pi at 7T [15,16]. Alternatively, black-blood properties of the stimulated echo single-voxel MRS (STEAM) with longer mixing time (TM) can be used at 7T to acquire cardiac spectra free of blood pool contamination [17].

In this study we sought to investigate the Pi/PCr in patients with T2DM using STEAM ^31^P-MRS at 7T and compare this metabolic parameter with healthy controls.

## Materials and methods

### Eligibility criteria and ethical considerations

All patients with diabetes and controls were over the age of 18, and were able to provide informed consent. Patients with diabetes were included if they had no change to their diabetic medication during the preceding three months and an HbA1C recorded between 6 and 9%. All study participants had a normal resting ECG and normal cardiovascular examination on the day of the study visit. Participants with any history of cardiovascular disease, hypertension, or renal impairment were excluded. Standard contraindications to MR at the 7T field strength applied to all. The study received ethical approval from the Oxford Research Ethics Committee (reference 13/SC/0376). All participants gave written informed consent prior to the study.

### Anthropomorphic, biochemical and clinical assessment

Height, weight and a resting electrocardiogram, blood pressure and heart were firstly recorded for all participants. A normal cardiovascular examination was also performed. In diabetics, fasting venous blood was taken for HBA1C, insulin, glucose, and a full lipid profile (unless these were available from a diabetic check occurring within the last month). Blood samples were analysed by the Oxford University Hospitals clinical biochemistry laboratory according to standardised protocols.

### Echocardiography

In patients with diabetes, echocardiography was performed on a Philips Vivid Q (Philips, Best, Netherlands) system to determine diastolic function; pulse wave velocity was measured at the mitral valve inflow to calculate E/A ratio, and tissue Doppler at the lateral and medial mitral valve annulus to generate E/e’ ratios, as well as the mean of medial and lateral measurements. Standard 2D examination of apical 2 and 4 chamber views were obtained and left ventricular ejection fraction (LVEF) determined by the biplane Simpson method.

### ^31^P magnetic resonance spectroscopy

All MR examinations were then performed on a human whole-body 7T Magnetom MR system (Siemens Healthineers, Erlangen, Germany). Single loop surface ^1^H transmit/receive coil (10 cm in diameter; Rapid Biomedical, Rimpar, Germany) was positioned over the heart of participants lying supine and used for cardiac localizers to guide ^31^P-MRS voxel placement and for shimming by a previously described [18] dedicated B_0_ shimming procedure. Briefly, a stack of 18 dual‐echo gradient‐recalled echo (GRE) slices aligned with the mid‐short‐axis view (TR 314 ms, TE_1_ 2.4 ms, TE_2_ 4.3 ms) was acquired to generate a B_0_ map. This field map was thresholded in MATLAB (MathWorks, Natick, MA, USA) by zeroing all pixels with an intensity less than that of the heart and setting the magnitude of all remaining pixels to 1. These modifications prevent the high‐intensity pixels near the surface dominating the vendor’s online computed magnitude-weighted shim solution.

^31^P-MRS was then performed after a coil change, using a 16-channel receive array combined with a ^31^P surface transmit coil (Rapid Biomedical) [18]. This coil comprises a rigid 27 x 28 cm^2^ transmit ^31^P element and a flexible array of 16 receive elements (size 8 x 5.5 cm^2^, arranged in a 4 x 4 grid). This configuration provides very high receive sensitivity from the heart, together with relatively homogeneous albeit rather weak transmit at the heart (peak B_1_^+^ was about 10 μT at the typical depth of the heart, i.e. 10 cm from the anterior chest wall) [17]. No respiratory triggering was used for ^31^P-MRS. Following previous work, cardiac gating was used for Pi/PCr measurements [17], but not for PCr/ATP measurements to save scan time [16,19].

First, the cardiac PCr/ATP ratio was measured using the approach of Ellis et al. [18], i.e. using an acquisition weighted 3D CSI with minimized acquisition delay (UTE-CSI) [20] with 8 × 16 × 6 matrix and field of view of 240 × 240 × 200 mm^3^, giving the nominal voxel size of 30 × 15 × 33.3 mm^2^, i.e. 15 mL. The CSI matrix was positioned with the 8 × 16 dimensions parallel to a cardiac short axis localizer and rotated in-plane so the phase-encoding direction with the highest resolution was in the anteroposterior direction to minimize skeletal muscle contamination. With TR of 2.2 s and 4 weighted averages in the centre of the k-space, the total acquisition time of the UTE-CSI was 6 minutes 40 seconds. RF excitation was performed using a shaped pulse that comprises a 0.5 ms hard pulse, preceded by a numerically optimized 1.9 ms part that improves homogeneity of excitation [21]. It excites an approximately 2 kHz bandwidth, hence, the centre excitation frequency was set to +266 Hz relative to PCr, as to cover metabolites from 2,3‐diphosphoglycerate (2,3‐DPG) to γ‐ATP.

Next, the Pi/PCr was measured using the interleaved STEAM ^31^P-MRS acquisitions MRS [17] with the voxel positioned over the interventricular septum and its size individually adjusted to cover as much septum as possible avoiding contamination from skeletal muscles (average size 58 ± 12 mL). The reference voltage required to achieve the 90° flip angle in the STEAM voxel was calculated during the UTE-CSI acquisition based on a set of inversion recovery free induction decay scans (IR-FIDs) of fiducials mounted on the ^31^P coil as described previously [17]. The rest of the parameters was set as follows: TE = 13 ms, effective TR = 6 s, TM_PCr_ = 7 ms, TM_Pi_ = 60 ms, and 256 averages.

### Magnetic resonance spectroscopy data analysis

MRS data were analysed, after whitened singular value decomposition (WSVD) coil combination [22], using the open-source Matlab-based Oxford Spectroscopy Analysis (OXSA) [23] toolbox’s implementation of the time domain fitting AMARES routine [24]. To fit the UTE-CSI dataset; PCr, phosphodiesters and the two diphosphate-glycerate (2,3-DPG) peaks were fitted as single Lorentzians, while the γ while the were fitted as single LorentziananianorentzianPG) peaks were fonly PCr, Pi and phosphodiesters were present and fitted as single Lorentzians. The ‘PCr-interleaf’ spectrum was fitted before the ‘Pi-interleaf’ spectrum so that the algorithm fitting the Pi frequency could be constrained to search around 5.0 ± 0.8 ppm relative to PCr, the linewidths of Pi and PCr were set to be equal, and the determined PCr phase was used as a starting point for fitting the Pi phase to improve the stability of Pi fitting [17]. No residual signals after fitting were above the noise level. Given myocardial [Pi] is extremely low and poor SNR may impact our ability to identify its resonance reliably, data were included for analysis only if the previously defined data quality conditions were met, i.e. the Pi peak was clearly resolved (defined as SNR >2.5 and visually obvious) [17]. Cramér-Rao lower bounds (CRLBs) were also calculated for the STEAM measured PCr and Pi amplitudes.

All metabolite signals were corrected for partial saturation using literature relaxation times [15,19] and the PCr/ATP ratio (using γusing determined from UTE-CSI was also corrected for blood contamination, assuming that blood ATP is ~15% of the combined 2,3-DPG signals [25]. Intramyocardial pH was calculated using the Henderson-Hasselbalch equation using chemical shift between PCr and Pi [26]. Student t-test was used to determine statistically significant (p<0.05) differences between the subject groups. Linear Pearson correlation was used to determine relation between ^31^P parameters and diastolic dysfunction (described as E/e’).

## Results

### Study population

Seventeen patients with type two diabetes (3 females), and twenty-three healthy controls (9 females) were recruited. Patients with diabetes had a significantly higher BMI (27.1 ± 4.2 kg.m^2^ vs. BMI 24.1 ± 2.6 kg.m^2^, p<0.01), and were older (61 ± 7 years vs. 43 ± 16 years, p<0.01). Resting mean arterial pressure (calculated as [2 x diastolic blood pressure + systolic blood pressure] / 3) was not different between the two groups [93±8mmHg (diabetes) vs. 90±8 mmHg (controls), p = 0.14], whereas resting heart rate was higher in patients with diabetes (66±9bpm vs. 60±9bpm, p<0.05). All T2DM patients had a Hb1Ac over 6% (mean 7.2 ± 1.2%) while their LVEF was in the normal range (mean 59 ± 4%). However, their E/e’ (average) was already mildly elevated (mean 6.9 ± 2.0) indicating low grade diastolic dysfunction. Further baseline data is given in Table 1.

**Table 1:** Details on the study population characteristics and demographics data.

	T2DM	Control	P-value*
**Number**	17 (3F)	23 (9F)	
**Age (years)**	61.4 ± 6.8	43.0 ± 16.4	<0.01
**BMI (kg/m^2^)**	27.1 ± 4.2	24.1 ± 2.6	<0.01
**Heart rate (b/min)**	66 ± 9	60 ± 10	<0.05
**Systolic blood pressure (mmHg)**	135 ± 14	126 ± 12	<0.05
**Diastolic blood pressure (mmHg)**	73 ± 8	71 ± 7	0.53
**Mean arterial pressure (mmHg)**	93 ± 8	90 ± 8	0.14
**HOMA-IR**	9.2 ± 3.8	-	
**HBA1c (%)**	7.2 ± 1.2	-	
**Total Cholesterol (mmol/l)**	3.7 ± 0.9	-	
**LDL Cholesterol (mmol/l)**	2.2 ± 0.6	-	
***Medication History***			
**Metformin**	17 (100%)	0	
**Sulfonylurea**	5 (29%)	0	
**SGLT-2 inhibitor**	1 (6%)	0	
**ACE inhibitor**	5 (29%)	0	
**Mineralocorticoid antagonist**	0	0	
**Statin ± Ezetimibe**	11 (65%)	0	
**Aspirin**	3 (18%)	0	
**Beta blocker**	0	0	
**Calcium channel blocker**	0	0	
***Echocardiography***			
**LVEF (%)**	59.3 ± 4.2	-	
**E/A**	0.9 ± 0.2	-	
**E/e’ (medial)**	7.8 ± 2.3	-	
**E/e’ (lateral)**	5.9 ± 1.9	-	
**E/e’ (average)**	6.9 ± 2.0	-	

**Fig 1:**
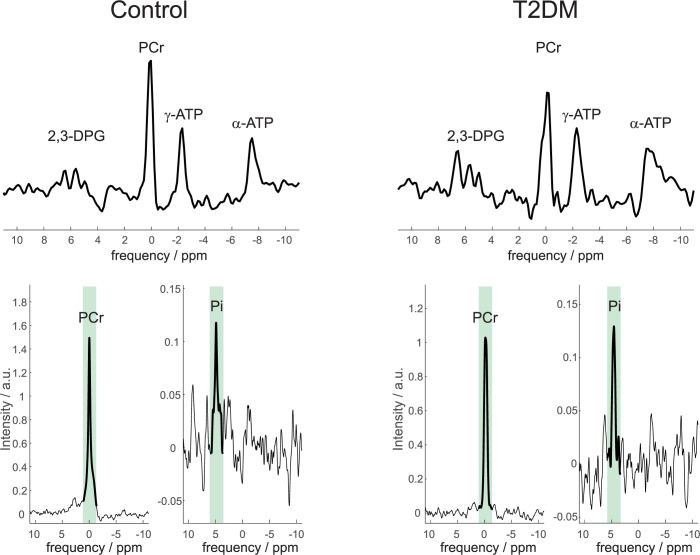
Typical CSI (top) and STEAM (bottom) spectra from a control (left) and a T2DM (right) participant. Please note that the CSI spectra are scaled to ATP amplitude to clearly demonstrate the difference in PCr/ATP. Also, for each STEAM interleave the spectral region targeted and used for the Pi/PCr and pH calculations is highlighted by bolder lines and green background. Increased Pi and decreased PCr can be seen in the T2DM STEAM data.

Due to myocardial [Pi] being low and BMI of some participants being well over 30 kg/m^2^, Pi was not reliably quantifiable in five T2DM and in one control STEAM spectra. The mean SNR of Pi and PCr was 6.5 ± 3.8 and 88 ± 41 for controls, and 6.7 ± 4.5 and 50 ± 30 for patients with diabetes, respectively. Hence the final comparison of cardiac Pi/PCr and myocardial pH was performed between 12 T2DM and 22 healthy participants. Similarly, a PCr/ATP measurement was not obtained in one diabetic and two controls due to poor data quality. Typical ^31^P-MRS spectra acquired in a control and a diabetic heart are depicted in Fig 1 both the UTE-CSI spectra used to determine PCr/ATP and the STEAM spectra of PCr and Pi acquired interleaved for the Pi/PCr quantification are depicted.

**Fig 2:**
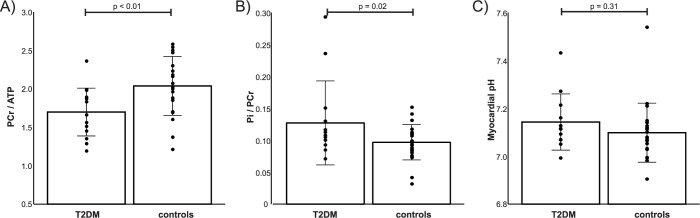
Energetics and myocardial pH in controls and diabetics. PCr/ATP was significantly lower (p<0.01, A) and Pi/PCr was significantly higher (p = 0.02, B) in T2DM patients. Myocardial pH was however not different (p = 0.31, C). Please note that due to myocardial [Pi] being low, and BMI of some participants being well over 30 kg/m^2^ Pi was reliably quantifiable [17], i.e. SNR>2.5 and clear peak, in 12/17 T2DM, and 22/23 control STEAM spectra.

**Fig 3:**
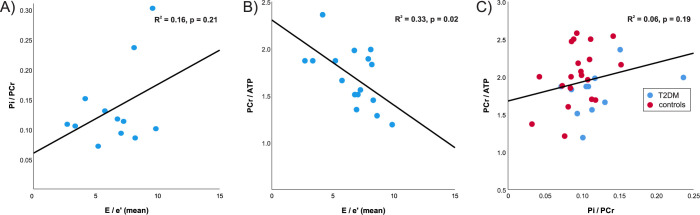
Correlation between cardiac metabolism and diastolic function in diabetic patients. E/e’ is an indicator of diastolic dysfunction with higher values indicating higher ventricular filling pressures. The parameter did not correlate with Pi/PCr (A) but did correlate inversely with PCr/ATP (B, p = 0.02) in the diabetics recruited. Overall, PCr/ATP did not correlate well with Pi/PCr but when plotted against one another (C) the shift to higher Pi/PCr and lower PCr/ATP in T2DM can be appreciated.

As expected, resting PCr/γ-ATP was significantly lower in diabetics compared to controls (1.70 ± 0.31 vs. 2.04 ± 0.38, p < 0.01). In the opposite direction and demonstrated for the first time here, cardiac Pi/PCr was significantly increased in T2DM patients (0.13 ± 0.07 vs. 0.10 ± 0.03, p = 0.02). The median (interquartile range) Pi CRLBs were 33.1% (22.9 to 50.3), and were not different between the two groups, i.e. 32.1% (23.5 to 50.2) vs 35.4% (22.4 to 58.3) for healthy controls and patients with diabetes, respectively. Fig 2 shows these differences in cardiac energetics together with the comparison of myocardial pH, which was not found to be significantly different between the participant groups (7.14 ± 0.12 in T2DM vs. 7.10 ± 0.12 in controls, p = 0.31). Within the diabetic group, E/e’ did not correlate with Pi/PCr (R^2^ = 0.16, p = 0.21, Fig 3A), but did negatively correlate with PCr/ATP (R^2^ = 0.33, p = 0.02, Fig 3B). Taking the cohort as a whole, no correlation was seen between the two metrics of cardiac energetics recorded directly, although, as can be appreciated in Fig 3C, patients with diabetes are shifted to lower PCr/ATP and higher Pi/PCr values.

## Discussion

We investigated cardiac inorganic phosphate reserves and myocardial pH in patients with type 2 diabetes mellitus, using *in vivo* ^31^P-MRS at 7T. Our results show that, as the PCr/ATP drops in T2DM patients, their cardiac Pi/PCr increases. At the same time, the myocardial pH does not differ between T2DM and healthy controls. Interestingly, we have found no correlation between PCr/ATP and Pi/PCr suggesting some level of mechanistic independence between them.

Our PCr/ATP result is in good agreement with literature, where the drop in PCr/ATP in diabetes has been extensively investigated [4,27,28]. Comparing the values of our measured PCr/ATP with literature reports, Levelt et al. [4] reported resting PCr/ATP of 1.74 ± 0.26 and 2.07 ± 0.35 and Scheuermann-Freestone et al. [27] reported 1.50 ± 0.11 and 2.30 ± 0.12 for T2DM patients and healthy controls, respectively. These are in good agreement with our measured values of 1.70 ± 0.31 and 2.07 ± 0.39 in T2DM and healthy controls, respectively.

The recently described long T_M_ STEAM at 7T technique allowed us to measure for the first time cardiac Pi/PCr in T2DM patients *in vivo*. We found significantly higher Pi/PCr values in the heart of T2DM patients in comparison to healthy controls, i.e. 0.13 ± 0.07 vs. 0.10 ± 0.03 (p = 0.02). This increase in Pi/PCr mirrors the drop in PCr/ATP discussed above, potentially limiting the available energy from ATP hydrolysis. If this progresses, diastolic dysfunction will progress and ultimately the systolic function will likely become impaired as well. Our reported values of Pi/PCr in healthy volunteers are in good agreement with literature reports, i.e. 0.10 ± 0.07 [14] or 0.10 ± 0.03 [17]. Similarly, the measured SNR and CRLB of the Pi signal are in good agreement with previous reports [17]. The Pi/PCr we measured for T2DM patients, while higher than those of healthy volunteers, are lower than those previously reported in patients with cardiomyopathies, i.e. 0.20 ± 0.08 [14] or 0.24 ± 0.09 [15]. This is in agreement with the reports on PCr/ATP where patients with cardiomyopathies often have even lower PCr/ATP than the patients with diabetes, putting cardiac metabolism of T2DM somewhere between healthy and failing heart [12]. This further suggests that slowly deteriorating cardiac energetics in T2DM could lead to diabetic cardiomyopathy and heart failure.

Alongside the Pi/PCr, we also report the first *in vivo* measured myocardial pH in T2DM patients. Our results show that the myocardial pH of T2DM patients is not statistically different than that of healthy controls, i.e. 7.14 ± 0.12 vs. 7.10 ± 0.12 (p = 0.31). This is in agreement with previous literature reports assessing myocardial pH in patients with cardiomyopathies, where similar values of 7.06 ± 0.03 [29] or 7.12 ± 0.04 [15] were shown in healthy volunteers, in comparison to 7.07 ± 0.02 [29] or 7.14 ± 0.02 [15] found in patients with hypertrophic cardiomyopathy. Broadband heteronuclear decoupling or long TR acquisition techniques were employed to measure myocardial pH in those studies. This shows that intracellular pH is robustly controlled and maintained in the diabetic myocardium at rest.

We did not find a correlation between diastolic dysfunction and the cardiac Pi/PCr (R^2^ = 0.16, p = 0.21) in T2DM in this study. On the other hand, E/e’ (mean) did negatively correlate with the cardiac PCr/ATP (R^2^ = 0.33, p = 0.02). This is in agreement with a recent study showing negative correlation between cardiac PCr/ATP and E/e’ over several stages of heart failure with preserved ejection fraction including T2DM [30], but not in T2DM only as showed in this study. In agreement with this, we did not observe any correlation (p = 0.19) between cardiac PCr/ATP and Pi/PCr. This potentially suggests that the rise in Pi may be mechanistically independent to the fall in PCr/ATP, with candidates including impairment of CK flux and substrate inflexibility [31].

There was a difference in age, BMI and systolic blood pressure between the two groups in our study, which could have confounded our results and could potentially be considered a limitation of our study. Also, due to the high BMI of several patients, significantly increasing the distance between the coil and the septum, the SNR of the Pi peak was too low to allow reliable Pi/PCr quantification in 5 out of 17 recruited patients. The use of dedicated higher B_1_^+^ performance RF coils, e.g., quadrature [32,33], or a birdcage design [34] combined with a similarly or more sensitive receive array as used here [35,36] could improve the achievable spectral SNR in such high BMI patients in the future. While we used a dedicated B_0_ shimming procedure, additional higher order shimming might improve the field homogeneity further and yield narrower linewidth and thus higher SNR. In addition, linewidth and SNR could in the future be also potentially improved through respiratory gating of the spectral acquisition.

Comparing metabolite ratios like PCr/ATP and Pi/PCr inherently depends on changes in both metabolites. While ATP concentration is considered to be stable in all but the most extreme conditions (such as acute ischemia), PCr content is more sensitive to change. Hence, it is unclear whether the observed Pi/PCr increase represents only a decrease in PCr or also an increase in cardiac Pi. In addition, our patient group was relatively small. Hence, further investigation of cardiac Pi metabolism in a larger cohort of patients with T2DM using recent absolute quantification techniques [37] will be needed to provide more definitive understanding of cardiac metabolism.

In Conclusion, using STEAM ^31^P-MRS at 7T we have observed for the first time increased Pi/PCr in the diabetic human heart in comparison to healthy subjects. While spectral SNR proved to be a limiting factor in patients with high BMI, good quality data were acquired in 12/17 patients. PCr/ATP and Pi/PCr were not significantly correlated, which hints that multiple mechanisms might contribute to these perturbations.

## References

[1] Poirier P, Bogaty P, Garneau C, Marois L, Dumesnil JG. Diastolic dysfunction in normotensive men with well-controlled type 2 diabetes: importance of maneuvers in echocardiographic screening for preclinical diabetic cardiomyopathy. Diabetes Care. 2001;24(1):5–10. doi: 10.2337/diacare.24.1.5 11194240

[2] Rubler S, Dlugash J, Yuceoglu YZ, Kumral T, Branwood AW, Grishman A. New type of cardiomyopathy associated with diabetic glomerulosclerosis. Am J Cardiol. 1972;30(6):595–602. doi: 10.1016/0002-9149(72)90595-4 4263660

[3] From AM, Scott CG, Chen HH. The development of heart failure in patients with diabetes mellitus and pre-clinical diastolic dysfunction a population-based study. J Am Coll Cardiol. 2010;55(4):300–5. doi: 10.1016/j.jacc.2009.12.003 20117433 PMC3878075

[4] Levelt E, Rodgers CT, Clarke WT, Mahmod M, Ariga R, Francis JM, et al. Cardiac energetics, oxygenation, and perfusion during increased workload in patients with type 2 diabetes mellitus. European heart journal. 2016;37(46):3461–9. doi: 10.1093/eurheartj/ehv442 26392437 PMC5201143

[5] Tan Y, Zhang Z, Zheng C, Wintergerst KA, Keller BB, Cai L. Mechanisms of diabetic cardiomyopathy and potential therapeutic strategies: preclinical and clinical evidence. Nat Rev Cardiol. 2020;17(9):585–607. doi: 10.1038/s41569-020-0339-2 32080423 PMC7849055

[6] Heather LC, Clarke K. Metabolism, hypoxia and the diabetic heart. J Mol Cell Cardiol. 2011;50(4):598–605. doi: 10.1016/j.yjmcc.2011.01.007 21262230

[7] Chong CR, Clarke K, Levelt E. Metabolic Remodeling in Diabetic Cardiomyopathy. Cardiovasc Res. 2017;113(4):422–30. doi: 10.1093/cvr/cvx018 28177068 PMC5412022

[8] Rider OJ, Apps A, Miller J, Lau JYC, Lewis AJM, Peterzan MA, et al. Noninvasive In Vivo Assessment of Cardiac Metabolism in the Healthy and Diabetic Human Heart Using Hyperpolarized (13)C MRI. Circ Res. 2020;126(6):725–36. doi: 10.1161/CIRCRESAHA.119.316260 32078413 PMC7077975

[9] Randle PJ, Garland PB, Hales CN, Newsholme EA. The glucose fatty-acid cycle. Its role in insulin sensitivity and the metabolic disturbances of diabetes mellitus. Lancet. 1963;1(7285):785–9. doi: 10.1016/s0140-6736(63)91500-9 13990765

[10] Bottomley PA, Charles HC, Roemer PB, Flamig D, Engeseth H, Edelstein WA, et al. Human in vivo phosphate metabolite imaging with 31P NMR. Magnetic resonance in medicine. 1988;7(3):319–36. doi: 10.1002/mrm.1910070309 3205148

[11] Valkovič L, Chmelík M, Krššák M. In-vivo(31)P-MRS of skeletal muscle and liver: A way for non-invasive assessment of their metabolism. Anal Biochem. 2017;529:193–215. doi: 10.1016/j.ab.2017.01.018 28119063 PMC5478074

[12] Watson WD, Miller JJJ, Lewis A, Neubauer S, Tyler D, Rider OJ, et al. Use of cardiac magnetic resonance to detect changes in metabolism in heart failure. Cardiovasc Diagn Ther. 2020;10(3):583–97. doi: 10.21037/cdt.2019.12.13 32695639 PMC7369287

[13] Rider OJ, Francis JM, Ali MK, Holloway C, Pegg T, Robson MD, et al. Effects of catecholamine stress on diastolic function and myocardial energetics in obesity. Circulation. 2012;125(12):1511–9. doi: 10.1161/CIRCULATIONAHA.111.069518 22368152

[14] Jung WI, Sieverding L, Breuer J, Hoess T, Widmaier S, Schmidt O, et al. 31P NMR spectroscopy detects metabolic abnormalities in asymptomatic patients with hypertrophic cardiomyopathy. Circulation. 1998;97(25):2536–42. doi: 10.1161/01.cir.97.25.2536 9657474

[15] Valkovič L, Clarke WT, Schmid AI, Raman B, Ellis J, Watkins H, et al. Measuring inorganic phosphate and intracellular pH in the healthy and hypertrophic cardiomyopathy hearts by in vivo 7T (31)P-cardiovascular magnetic resonance spectroscopy. J Cardiovasc Magn Reson. 2019;21(1):19. doi: 10.1186/s12968-019-0529-4 30871562 PMC6419336

[16] Wampl S, Korner T, Valkovič L, Trattnig S, Wolzt M, Meyerspeer M, et al. Investigating the effect of trigger delay on cardiac 31P MRS signals. Sci Rep. 2021;11(1):9268. doi: 10.1038/s41598-021-87063-8 33927234 PMC8085231

[17] Apps A, Valkovič L, Peterzan M, Lau JYC, Hundertmark M, Clarke W, et al. Quantifying the effect of dobutamine stress on myocardial Pi and pH in healthy volunteers: A (31) P MRS study at 7T. Magn Reson Med. 2021;85(3):1147–59. doi: 10.1002/mrm.28494 32929770 PMC8239988

[18] Ellis J, Valkovič L, Purvis LAB, Clarke WT, Rodgers CT. Reproducibility of human cardiac phosphorus MRS ((31) P-MRS) at 7 T. NMR Biomed. 2019;32(6):e4095. doi: 10.1002/nbm.4095 30924566 PMC6546607

[19] Rodgers CT, Clarke WT, Snyder C, Vaughan JT, Neubauer S, Robson MD. Human cardiac 31P magnetic resonance spectroscopy at 7 Tesla. Magnetic resonance in medicine. 2014;72(2):304–15. doi: 10.1002/mrm.24922 24006267 PMC4106879

[20] Robson MD, Tyler DJ, Neubauer S. Ultrashort TE chemical shift imaging (UTE-CSI). Magnetic resonance in medicine. 2005;53(2):267–74. doi: 10.1002/mrm.20344 15678544

[21] Tyler DJ, Emmanuel Y, Cochlin LE, Hudsmith LE, Holloway CJ, Neubauer S, et al. Reproducibility of 31P cardiac magnetic resonance spectroscopy at 3 T. NMR Biomed. 2009;22(4):405–13. doi: 10.1002/nbm.1350 19023865

[22] Rodgers CT, Robson MD. Receive array magnetic resonance spectroscopy: Whitened singular value decomposition (WSVD) gives optimal Bayesian solution. Magn Reson Med. 2010;63(4):881–91. doi: 10.1002/mrm.22230 20373389

[23] Purvis LAB, Clarke WT, Biasiolli L, Valkovič L, Robson MD, Rodgers CT. OXSA: An open-source magnetic resonance spectroscopy analysis toolbox in MATLAB. PloS one. 2017;12(9):e0185356. doi: 10.1371/journal.pone.0185356 28938003 PMC5609763

[24] Vanhamme L, van den Boogaart A, Van Huffel S. Improved method for accurate and efficient quantification of MRS data with use of prior knowledge. Journal of magnetic resonance. 1997;129(1):35–43. doi: 10.1006/jmre.1997.1244 9405214

[25] Horn M, Neubauer S, Bomhard M, Kadgien M, Schnackerz K, Ertl G. 31P-NMR spectroscopy of human blood and serum: first results from volunteers and patients with congestive heart failure, diabetes mellitus and hyperlipidaemia Magn Reson Mater Phy. 1993;1(2):55–60.

[26] Bailey IA, Williams SR, Radda GK, Gadian DG. Activity of phosphorylase in total global ischaemia in the rat heart. A phosphorus-31 nuclear-magnetic-resonance study. Biochem J. 1981;196(1):171–8. doi: 10.1042/bj1960171 7306067 PMC1162979

[27] Scheuermann-Freestone M, Madsen PL, Manners D, Blamire AM, Buckingham RE, Styles P, et al. Abnormal cardiac and skeletal muscle energy metabolism in patients with type 2 diabetes. Circulation. 2003;107(24):3040–6. doi: 10.1161/01.CIR.0000072789.89096.10 12810608

[28] Diamant M, Lamb HJ, Groeneveld Y, Endert EL, Smit JW, Bax JJ, et al. Diastolic dysfunction is associated with altered myocardial metabolism in asymptomatic normotensive patients with well-controlled type 2 diabetes mellitus. J Am Coll Cardiol. 2003;42(2):328–35. doi: 10.1016/s0735-1097(03)00625-9 12875772

[29] Sieverding L, Jung WI, Breuer J, Widmaier S, Staubert A, van Erckelens F, et al. Proton-decoupled myocardial 31P NMR spectroscopy reveals decreased PCr/Pi in patients with severe hypertrophic cardiomyopathy. The American journal of cardiology. 1997;80(3A):34A–40A. doi: 10.1016/s0002-9149(97)00456-6 9293954

[30] Burrage MK, Hundertmark M, Valkovič L, Watson WD, Rayner J, Sabharwal N, et al. Energetic Basis for Exercise-Induced Pulmonary Congestion in Heart Failure With Preserved Ejection Fraction. Circulation. 2021;144(21):1664–78. doi: 10.1161/CIRCULATIONAHA.121.054858 34743560 PMC8601674

[31] Rider OJ, Apps A, Miller JJ, Lau JY, Lewis AJ, Peterzan MA, et al. Non-Invasive In Vivo Assessment of Cardiac Metabolism in the Healthy and Diabetic Human Heart Using Hyperpolarized (13)C MRI. Circ Res. 2020. 10.1161/CIRCRESAHA.119.316260 PMC7077975 32078413

[32] Schaller B, Paritmongkol W, Rodgers CT, editors. Quadrature 31P and single 1H dual-tune coil for cardiac 31P-MRS at 7T. 24th Annual Meeting of ISMRM; 2016; Singapore, Singapore.

[33] Giovannetti G, Frijia F, Hartwig V, Attanasio S, Menichetti L, Vanello N, et al. Design of a quadrature surface coil for hyperpolarized 13C MRS cardiac metabolism studies in pigs. Concept Magn Reson B. 2013;43b(2):69–77.

[34] Loring J, van der Kemp WJ, Almujayyaz S, van Oorschot JW, Luijten PR, Klomp DW. Whole-body radiofrequency coil for (31) P MRSI at 7 T. NMR Biomed. 2016;29(6):709–20. doi: 10.1002/nbm.3517 27037615

[35] Valkovič L, Dragonu I, Almujayyaz S, Batzakis A, Young LAJ, Purvis LAB, et al. Using a whole-body 31P birdcage transmit coil and 16-element receive array for human cardiac metabolic imaging at 7T. PloS one. 2017;12(10):e0187153. doi: 10.1371/journal.pone.0187153 29073228 PMC5658155

[36] Froeling M, Prompers JJ, Klomp DWJ, van der Velden TA. PCA denoising and Wiener deconvolution of (31) P 3D CSI data to enhance effective SNR and improve point spread function. Magn Reson Med. 2021;85(6):2992–3009. doi: 10.1002/mrm.28654 33522635 PMC7986807

[37] Purvis LAB, Valkovič L, Robson MD, Rodgers CT. Feasibility of absolute quantification for 31P MRS at 7 T. Magn Reson Med. 2019;82(1):49–61. doi: 10.1002/mrm.27729 30892732 PMC6492160

